# The Prevalence of Inguinal Hernia Among Athletes in Saudi Arabia: A Cross-Sectional Study

**DOI:** 10.7759/cureus.34466

**Published:** 2023-01-31

**Authors:** Abdullah S Alqahtani, Mohammed Eid M Alotaibi, Imtenan A Oberi, Lana R Alrashidi, Malik M Alreshidi, Jana K Abukhlaled

**Affiliations:** 1 General Surgery, King Saud Medical City, Riyadh, SAU; 2 College of Medicine, Imam Mohammad Ibn Saud Islamic University, Riyadh, SAU; 3 Collage of Medicine, Jazan University, Jazan City, SAU; 4 College of Medicine, Unaizah College of Medicine and Medical Sciences, Qassim University, Unaizah, SAU; 5 College of Medicine, Taibah University, Medina, SAU; 6 College of Medicine, Princess Nourah bint Abdulrahman University, Riyadh, SAU

**Keywords:** groin injury, athletics, athletic pubalgia, weightlifting, sports-related, ksa, saudi arabia, athletes, inguinal hernia

## Abstract

Introduction

An inguinal hernia is the most common type of hernia. It might manifest as a groin bulge, lump, or enlarged scrotum. Swelling may be uncomfortable and painful and even cause intestinal obstruction. This study aimed to measure the prevalence of inguinal hernia among athletes in Saudi Arabia.

Subject and methods

This is a cross-sectional study conducted among Saudi Arabian athletes. A self-administered questionnaire was distributed among athletes using an online survey through different Saudi Olympic Training and Fitness Centers throughout the kingdom. The questionnaire includes sociodemographic characteristics (i.e. age, gender, etc.), risk factors, and complications of inguinal hernia.

Results

Of the 594 athletes, 55.6% were females and 57.6% were aged between 18 and 24 years. The most common type of sport was running (31%). The most common risk factor for inguinal hernia was previous abdominal surgery (57.5%). The prevalence of inguinal hernia among Saudi athletes was 12.3%. Being older in age and being male were the independent significant predictors associated with increased risk for inguinal hernia, whereas weightlifting was the independent significant factor of decreased risk for inguinal hernia.

Conclusion

The prevalence of inguinal hernia among athletes was 12.3%. Older male athletes were most likely at a greater risk to suffer from inguinal hernia as compared to the rest of the athletes. Further research is needed to extract more data about the prevalence of inguinal hernia among Saudi Arabian athletes and determine its risk factors.

## Introduction

The most common type of hernia is an inguinal hernia. It might manifest as a groin bulge, lump, or enlarged scrotum. Swelling may be uncomfortable and painful, and even cause intestinal obstruction [1]. The lump usually bulges while lifting anything and goes away when lying down. An inguinal hernia often develops near the top of the inner thigh when fatty tissue or a portion of the bowel, such as the intestine, pushes through into the groin, and it enters a region known as the inguinal canal by pushing through a weak area in the surrounding muscle wall.

Inguinal hernia repair is one of the most commonly performed surgery worldwide [2]. It affects 220 million individuals and is one of the most prevalent surgical procedures globally. Most inguinal hernia cases are seen in men [3]. Risk factors include straining on the toilet while constipating or pulling large weights, as these actions create pressure on the abdomen and can cause inguinal hernias to emerge abruptly.

Inguinal hernia problems cause approximately 40,000 fatalities annually and a loss of 3,500,000 years of life due to incapacity [4]. An inguinal hernia may result in obstruction in which a portion of the intestine gets stuck in the inguinal canal and cause pain in the groin, nausea, and vomiting. Additionally, it can cause strangulation. During a case of strangulation, a portion of the intestine gets caught, cutting off its blood supply; this situation necessitates immediate emergency surgery to free the trapped tissue and re-establish its blood supply so that it does not perish.

However, an inguinal hernia might develop from a sports hernia [5]. A sports hernia, also known as athletic pubalgia, is a disorder characterized by persistent supra-inguinal groin discomfort brought by activity that is connected to a developing direct inguinal wall bulge whenever the abdominal muscles contract strongly.

Since these disorders are relatively frequent among athletes, excessive physical activity is considered the etiological cause. A more significant risk exists among athletes with a history of groin injuries, older athletes, players who take off time from competition, and athletes who specialize in sports that use only a portion of their muscles [6]. Inguinal hernia is a common source of pain in athletes [7]. 

Our study aims to fill the knowledge gaps regarding the prevalence and possible risk factors for hernias among athletes in Saudi Arabia.

## Materials and methods

This cross-sectional study was performed among athletes diagnosed with inguinal hernias in Saudi Arabia. All Saudi male and female athletes above the age of 18 who had been diagnosed with inguinal hernia were included in the study. On the other hand, non-Saudis, non-athletes, and individuals under 18 were not included in this research. The participants were randomly selected from athletes diagnosed with an inguinal hernia by physicians in Saudi Arabia between September 25 and November 25, 2022. The sample size was determined using the Raosoft sample size calculator (Raosoft Inc., Seattle, Washington, United States), and a total of 385 athletes were required to achieve a 95% confidence interval with a 5% margin of error.

Data collection tools and method

We used a self-structured Arabic questionnaire consisting of three sections, including an informed consent page with all the information about the study. The first section of the questionnaire was for sociodemographic information, the second section was for the past medical history of the participants, including a list of risk factors for developing an inguinal hernia, and the last section was for detailed information about the hernia.

Our questionnaire was drafted in English and then translated into Arabic by two independent experts. One was outside the medical field and the second was in the medical field. The questionnaire was validated first by expert evaluation. Content validity and face validity were established by the expert's evaluation. Then a pilot study was done that included 38 responses. We evaluated the internal consistency using Cronbach's α coefficient. A coefficient of 0.7 and above was indicative of good internal consistency.

The questionnaire was distributed online using Google Forms (Google LLC, Menlo Park, California, United States) and sent to the Saudi Olympic Training Centers around the kingdom, in addition to fitness centers. All participants were informed that no indentification would be necessary and that the data would be kept secure with only authorized access.

Ethical considerations

The Internal Review Board for Ethics in Research on Living Creatures at Imam Muhammad bin Saud Islamic University, Riyadh, Saudi Arabia approved the study (Approval number: 323/2022)

Statistical analysis

Descriptive statistics were summarized as numbers and percentages for all categorical variables. The prevalence of inguinal hernia was compared with the sociodemographic characteristics of the athletes by using the Chi-square test. Significant results were then gathered and tested in a multivariate regression model to determine the independent significant factors associated with inguinal hernia where the odds ratio, as well as 95% confidence interval, were also reported. A P-value of 0.05 was considered statistically significant. All data analyses were performed using IBM SPSS Statistics for Windows, Version 26.0 (Released 2019; IBM Corp., Armonk, New York, United States).

## Results

**Figure 1:**
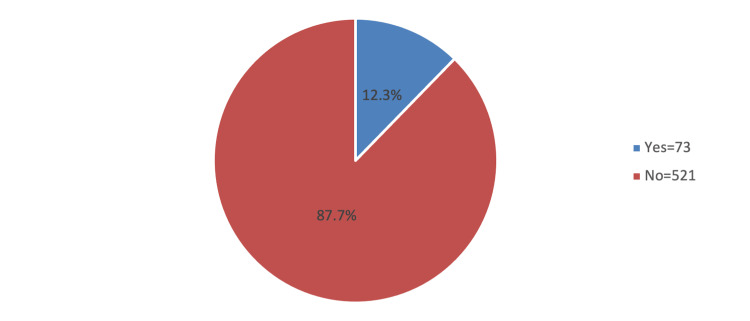
Prevalence of inguinal hernia among athletes

A total of 594 athletes were involved; 57.6% were aged between 18 and 24 years with more than half (55.6%) being females. Table 1 presents the sociodemographic characteristics of the athletes. Approximately 43.8% were living in the western region. The most common type of sport associated with the athletes was running (31%), followed by weightlifting (29.8%) with 38.2% practicing the sport for one to two hours per week. The prevalence of inguinal hernia among the study participants was 12.3% (Figure 1).

**Table 1:** Sociodemographic characteristics of the athletes (n=594)

Study variables	N (%)
Age group	
18-24 years	342 (57.6%)
25-34 years	163 (27.4%)
35-44 years	54 (09.1%)
≥45 years	35 (05.9%)
Gender	
Male	264 (44.4%)
Female	330 (55.6%)
Region of Residence	
Northern Region	46 (07.7%)
Western Region	260 (43.8%)
Central Region	62 (10.4%)
Eastern Region	58 (09.8%)
Southern Region	168 (28.3%)
Type of Sports	
Running	184 (31.0%)
Weightlifting	177 (29.8%)
Football	113 (19.0%)
Athletics	28 (04.7%)
Basketball and volleyball	26 (04.4%)
Boxing	22 (03.7%)
Other	44 (07.4%)
Number of Hours Per Week Spent Practicing This Sport	
1-2 hours per week	227 (38.2%)
3-4 hours per week	172 (29.0%)
5-7 hours per week	118 (19.9%)
>7 hours per week	77 (13.0%)

The characteristics of the athletes with inguinal hernia (n=73) are given in Table 2. Approximately three-quarters (75.3%) had been diagnosed with a hernia by a doctor in approximately the past five years or less. The proportions of athletes with a previous family history of hernia, previous abdominal surgery, previous history of abdominal trauma, chronic cough, constipation, and smoking history were 42.5%, 57.5%, 41.1%, 43.8%, 47.9%, and 45.2%, respectively. Additionally, the prevalence of athletes who complained of chronic pain during sports due to hernia was 54.8%. Of those who complained of pain (n=40), more than half (54.8%) complained of pain when touching the area of the hernia, 46.6% indicated the size of the hernia is moderate, and 83.6% reported that the hernia was reducible.

**Table 2:** Risk factors and complications of inguinal hernia among athletes (n=73)

Variables	N (%)
How Were You Diagnosed with a Hernia?	
By physician	55 (75.3%)
Self-diagnosed	08 (11.0%)
Seen by family members	06 (08.2%)
By imaging studies (US, MRI, CT scan)	04 (05.5%)
When Was The Time of Diagnosis?	
<1 year ago	23 (31.5%)
1-2 years ago	23 (31.5%)
3-4 years ago	10 (13.7%)
≥5 years ago	17 (23.3%)
Do You Have Any Family History of Hernia?	
Yes	31 (42.5%)
No	42 (57.5%)
Do You Have Any Previous Abdominal Surgery?	
Yes	42 (57.5%)
No	31 (42.5%)
Do You Have Any History of Abdominal Trauma?	
Yes	30 (41.1%)
No	43 (58.9%)
Do You Have a Chronic Cough?	
Yes	32 (43.8%)
No	41 (56.2%)
Do You Have Chronic Constipation?	
Yes	35 (47.9%)
No	38 (52.1%)
Do You Smoke?	
Yes	33 (45.2%)
No	40 (54.8%)
Do You Complain of Chronic Pain During Sports Because of The Hernia?	
Yes	40 (54.8%)
No	33 (45.2%)
Do You Complain of Any Pain When Touching the Area?	
Yes	40 (54.8%)
No	33 (45.2%)
What is The Size of The Hernia?	
Small	20 (27.4%)
Moderate	34 (46.6%)
Huge	05 (06.8%)
Not seen	14 (19.2%)
Is The Hernia Reducible?	
Yes	61 (83.6%)
No	12 (16.4%)
Do You Have Any Complications?	
Yes	32 (43.8%)
No	41 (56.2%)
Surgical Correction of the Hernia?	
Yes	45 (61.6%)
No	28 (38.4%)

We used the Chi-square test to measure the influence of inguinal hernia in terms of sociodemographic variables (Table 3). It was observed that the prevalence of inguinal hernia was significantly more common among the older age group (p<0.001), the male gender (p<0.001), and those who do boxing (p<0.001). 

**Table 3:** Factors that influence inguinal hernia among athletes (n=594) ^§^ P-value has been calculated using Chi-square test;   ** Significant at p<0.05 level.

Factor	Inguinal Hernia		P-value ^§^
	Yes N (%) ^(n=73)^	No N (%) ^(n=521)^	
Age group			
<25 years	50 (68.5%)	455 (87.3%)	<0.001 **
≥25 years	23 (31.5%)	66 (12.7%)	
Gender			
Male	48 (65.8%)	216 (41.5%)	<0.001 **
Female	25 (34.2%)	305 (58.5%)	
Type of Sports			
Weightlifting	18 (24.7%)	159 (30.5%)	<0.001 **
Football	15 (20.5%)	98 (18.8%)	
Athletics, and running	24 (32.9%)	188 (36.1%)	
Boxing	11 (15.1%)	11 (02.1%)	
Other	05 (06.8%)	65 (12.5%)	
Number of Hours per Week Spent For Practicing This Sport			
1-2 hours per week	22 (30.1%)	205 (39.3%)	0.280
3-4 hours per week	21 (28.8%)	151 (29.0%)	
5-7 hours per week	20 (27.4%)	98 (18.8%)	
>7 hours per week	10 (13.7%)	67 (12.9%)	

In a multivariate regression model (Table 4), it was found that the older age group and male gender were the independent significant factors associated with an increased risk of inguinal hernia while weightlifting was the sole independent significant predictor of a decreased risk of inguinal hernia. This further suggests that compared to the younger age group, the risk of having an inguinal hernia was likely to increase among older athletes by at least 3.46 times (adjusted OR (AOR) 3.456; 95%CI 1.865-6.404; p<0.001). Compared to female athletes, male athletes were 2.88 times more likely to be associated with inguinal hernia (AOR 2.880; 95%CI 1.618-5.127; p<0.001). Finally, compared to athletes who were playing other sports such as basketball, volleyball, or swimming, the risk for inguinal hernia in athletes who regularly performed weightlifting was predicted to decrease by at least 85% (AOR 0.150; 95%CI 0.054-0.417; p<0.001).

**Table 4:** Multivariate regression analysis to determine the independent significant factors associated with inguinal hernia (n=594) ** Significant at p<0.05 level. AOR: adjusted odds ratio; CI: confidence interval

Factor	AOR	95% CI	P-value
Age Group			
<25 years	Ref		
≥25 years	3.456	1.865 – 6.404	<0.001 **
Gender			
Male	2.880	1.618 – 5.127	<0.001 **
Female	Ref		
Type of Sports			
Weightlifting	0.150	0.054 – 0.417	<0.001 **
Football	1.402	0.481 – 4.087	0.536
Athletics, and running	1.055	0.494 – 2.251	0.890
Boxing	0.934	0.467 – 1.869	0.934
Other	Ref		

## Discussion

This study investigated the prevalence of inguinal hernia among Saudi Arabian athletes. The prevalence of inguinal hernia among athletes was 12.3%. This prevalence is consistent with the report of Ohene-Yeboah et al. [8]. The prevalence of untreated inguinal hernia among adult men in Ghana was 10.8% while in a study by Williamson et al. [9], 14 % of patients reported a single strenuous event (SSE) concomitant with the onset of hernia. Of them, only 5% reporting a hernia associated with SSE met published criteria for the association of the hernia with SSE, representing less than 1% of all patients treated for inguinal hernia in a one-year period at a single center. However, in the Arar region, among patients with an abdominal hernia (11.7%), the prevalence of inguinal hernia cases was 27.3%, and other detected cases were para-umbilical hernia (33.9%) and umbilical hernia (20.8%) [10]. In Brazil, the presence of an inguinal hernia was detected in 20.9% of patients [11], which was higher than our report. More investigation is required to ascertain the prevalence range of inguinal hernia among individuals who are engaged in strenuous activities.

The risk of inguinal hernia was more associated with the older age group (age ≥25 years). This result has also been proven across the literature wherein increasing age is correlated with an increased risk for inguinal hernia [8,12-14]. Khalaf also documented that even though most of the patients were aged more than 40 years, their BMI was estimated to be in the normal range and most of the patients had chosen hernia repair. where 12% developed postoperative complications [14]. In our study, although the majority were in the normal BMI range, however, this did not reach a statistical significance (p=0.441).

Likewise, in our multivariate regression estimates, male athletes were predicted to have an approximately three times higher risk of inguinal hernia than female athletes. This mirrored the studies conducted by AhmedAlenazi et al. [10] and Iqbal et al. [15]. Both studies reported that the prevalence of hernia-related disease was higher in males than in females. In Sierra Leone, reports indicated that the major burden for the male population in Sierra Leone was groin masses [16]. Hence, citing problems with the cost, the author concluded that improving access to surgical care for adult patients with hernias and early intervention for children are needed to address the burden of disease and prevent complications or limitations of daily activity.

Despite a positive association between inguinal hernia among gender and age, in terms of the type of sports, our results suggest that compared to athletes who played other sports (i.e. swimming, basketball, and volleyball), the risk of inguinal hernia among athletes who were engaged in weightlifting was significantly less likely. However, a greater effect was seen in athletes who played football even though the effect of football on inguinal hernia in the adjusted model did not reach statistical significance (p=0.536). Thus, this result is subjected to further investigation to determine the true effect of the type of sports in relation to inguinal hernia. In Nigeria [17], research revealed that the significant predictors of inguinal hernia were strenuous work activities and a positive history of hernia. These findings were also shown in our results. Accordingly, we established that family history (42.5%), previous abdominal surgery (57.5%), history of abdominal trauma (41.1%), chronic cough (43.8%), chronic constipation (47.9%), and smoking (45.2%) were risk factors for inguinal hernia in athletes. Respondents living in the Al-jouf region seem to have a better awareness of the risk factors for developing a hernia [18]. Based on the reports, the subjects knew that heavy lifting (89.5%), pregnancy (88.5%), previous surgery (86%), constipation (81%), asthma (59%), enlarged prostate (41%), and smoking (40%) were all risk factors for a hernia, and their overall knowledge regarding hernia risk factors was either very good (38%) or good (36%).

Conversely, among athletes with inguinal hernia, the prevalence of those who suffered complications due to inguinal hernia was 43.8% and that of those who complained of pain during sports was 54.8%. Hence, 61.6% of them underwent surgical intervention for the management of the disease. This is almost consistent with the study of AhmedAlenazi et al. in which 20.2% experienced complications [10]. Approximately 47.5% of patients underwent surgical procedures while others preferred conservative treatment instead (47%). In Brazil, patients' treatment duration ranged from one month to 12 months; however, the rates of patients who came back to play sports were relatively higher at 95.2% [11]. These numbers concurred with the study done in Italy [19]. The study documented that the duration to return to full sports activity was approximately one month (94.4%) and at 9 months, 98.5% of the patients were active. On 13 years of follow-up, the study reported a recurrence rate of 2.5%.

Limitation

There is a potential for recall bias in our survey as the data were collected through self-reported questionnaire.

## Conclusions

The prevalence of inguinal hernia among athletes was 12.3%. Older male athletes were most likely at a greater risk to suffer from inguinal hernia as compared to the rest of the athletes. The initiatives of government Olympic centers are imperative to monitor those athletes who were at greater risk for developing hernias. This may help in early detection and treatment; thereby reducing the prevalence and worst-case scenario of this condition. Further research is needed to extract more data about the prevalence of inguinal hernia among Saudi Arabian athletes and determine its risk factors.
